# SARS-CoV-2 Nsp1 suppresses the canonical NF-κB pathway by promoting ubiquitin-dependent degradation of TAK1 kinase

**DOI:** 10.1371/journal.ppat.1014191

**Published:** 2026-05-04

**Authors:** Han-cheng Wei, Qingxin Yang, Hong Yang, Yu-hang Wang, Kai Wang, Kepan Linghu, Natacha S. Ogando, Xiaoya Huang, Eric J. Snijder, Yu Zhong, Yu Chen, Quan Yuan, Lu Chen, Jing-wen Lin

**Affiliations:** 1 Center of Infectious Diseases and State Key Laboratory of Biotherapy, West China Hospital, Sichuan University, Chengdu, Sichuan, China; 2 Center for Biological and Translational Research, Biosafety Laboratory of West China Hospital, West China Hospital, Sichuan University, Chengdu, Sichuan, China; 3 State Key Laboratory of Vaccines for Infectious Diseases, Xiang An Biomedicine Laboratory, School of Public Health & School of Life Sciences, Xiamen University, Xiamen, China; 4 Molecular Virology Laboratory, Leiden University Center for Infectious Diseases (LUCID), Leiden University Medical Center, Leiden, The Netherlands; 5 State Key Laboratory of Virology, RNA Institute, College of Life Sciences and Frontier Science Center for Immunology and Metabolism, Wuhan University, Wuhan, China; 6 Key Laboratory of Birth Defects and Related Diseases of Women and Children of MOE, Department of Laboratory Medicine, West China Second University Hospital, Sichuan University, Chengdu, China; Washington University School of Medicine in Saint Louis: Washington University in St Louis School of Medicine, UNITED STATES OF AMERICA

## Abstract

Immunoregulatory proteins expressed by SARS-CoV-2 interfere with host antiviral defences in infected cells and play critical roles in the pathogenesis and clinical manifestations of COVID-19. Here, we established a prediction algorithm by integrating a pretrained protein-language model and gene weights in immune-related pathways to quantify perturbations of SARS-CoV-2 proteins in host immunity. The results revealed that the canonical NF-κB pathway was dynamically regulated by SARS-CoV-2 infection and that nonstructural protein 1 (Nsp1) significantly suppressed the activation of the NF-κB pathway by other viral proteins and proinflammatory cytokines, such as IL-1β. Nsp1 binds to TAK1 at the TAB1-binding domain, promoting TRIM21-mediated K48-linked ubiquitination and subsequent proteasomal protein degradation, leading to the inactivation of the NF-κB signalling pathway. This work presents a novel framework to identify viral immunoregulators at the pathway level and provides mechanistic insights into immune evasion by SARS-CoV-2.

## Author summary

SARS-CoV-2 infection causes a clinical spectrum ranging from asymptomatic infection to fatal illness, in which the dysregulated immune response plays a role. Unravelling the mechanisms of immunoregulation by viral proteins would aid in the development of new immunotherapies. In this study, we established a deep-learning algorithm to bridge the gap between ‘binary’ virus-host protein-protein interactions and complex pathway-level regulation. With the help of this predictor, GWPIS, we identified a key NF-κB suppressor, Nsp1. Mechanistically, Nsp1 binds to the TAB1-binding domain of TAK1, thereby inhibiting the formation of the TAK1-TAB1 complex and downstream signaling, including the NF-κB, MAPK and AP-1 pathways. Moreover, Nsp1 promotes TRIM21-dependent K48-linked polyubiquitination of TAK1, leading to proteasomal degradation and downregulation of TAK1, further suppressing downstream signaling. This study provides novel insights into how the virus manipulates the host ubiquitin machinery to suppress antiviral responses.

## Introduction

SARS-CoV-2 continues to pose a major global health threat because of its ability to rapidly evolve and evade host antiviral defences and the uncertainties surrounding the long-term efficacy of existing vaccines and therapeutics [1]. The manipulation of immune responses by SARS-CoV-2 is a complex process regulated by multiple viral proteins, including structural, nonstructural (Nsp) and accessory proteins. For example, open reading frame (ORF) 9b inhibits the RNA-sensing adaptor MAVS [2], and the N protein and Nsp5 prevent the activation of the RNA sensor RIG-I and subsequently suppress type I and type III interferon (IFN) responses [3]. In addition, Nsps, including Nsp1, Nsp3, Nsp5, Nsp6 and Nsp12, suppress IFN-I and impair early antiviral immune responses [4–7]. ORF6 inhibits the nuclear translocation of IRF3 and STAT1 [8,9] and subsequent antiviral signaling. In addition to IFNs, the NF-κB signaling pathway plays critical roles in proinflammatory responses [10]. Its hyperactivation leads to a cytokine storm characterized by elevated IL-6, TNF-α, and IL-1β levels, which drive acute respiratory distress syndrome (ARDS) and systemic organ dysfunction in severe COVID-19 [11,12]. Unlike the clear IFN-suppression effect exerted by SARS-CoV-2, the regulation of NF-κB is more complex, with many viral proteins reported to activate signaling [13–21], while others exert suppressive effects [16,22–24].

Identification of virus-host protein interactions is essential for understanding the molecular basis of viral pathogenesis and host defence [25]. Machine-learning models based on algorithms such as support vector machines (SVMs), random forests (RFs) or deep neural networks (DNNs) have been developed to identify protein-protein interactions (PPIs) by interrogating features such as gene expression, protein sequences, structures, domain-domain interactions and network topology [26,27]; methods have also been developed to identify host-pathogen PPIs [28]. To improve the prediction accuracy, deep learning methods have also emerged. Architectures such as convolutional neural networks (CNNs) and multilayer perceptrons (MLPs), which are effective at capturing complex patterns directly from sequence data with minimal or no manual curation, have been used in models of DeepViral [29], deepHPI [30], ProtInteract [31], and Deep-HPI-pred [32].

The immune response against pathogens is highly complex, and pathogen protein-mediated immunoregulation occurs in the context of a pathway or even a network. As a result, the existing methods focusing on the ‘binary’ interactions between the pathogen and host proteins provide limited results. To address this, we established a screening framework by integrating PPI prediction based on a pretrained protein language model D-SCRIPT [33] and gene weights in immune-related pathways by PROGENy [34], providing a geometric weighted pathway interaction score (GWPIS) to estimate the interference of pathogen proteins in host pathways. Here, we applied GWPIS to investigate the immunoregulation of SARS-CoV-2 in infected cells and identified Nsp1 as a key viral factor that suppresses NF-κB by interacting with TGF-β-activated kinase (TAK1) [35].

## Results

### Dynamic regulation of the canonical NF-κB pathway by SARS-CoV-2

**Fig 1:**
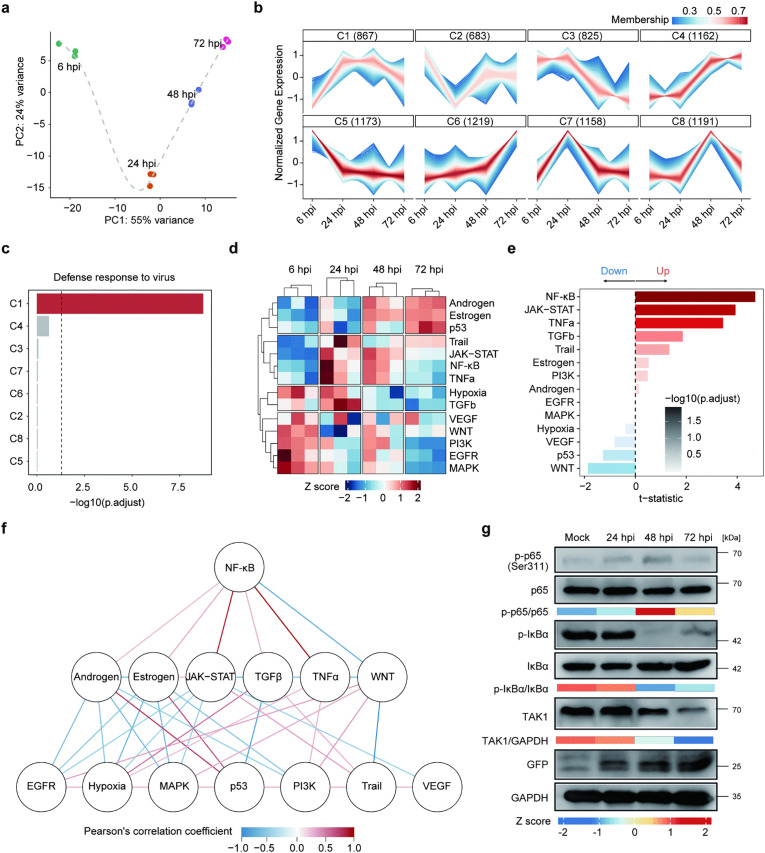
SARS-CoV-2 infection dynamically regulates viral defence pathways in infected host cells. **a**: PCA (principal component analysis) of the transcriptomes of Calu-3 cells infected with SARS-CoV-2 at an MOI of 2. Infected cells were collected at 6, 24, 48 and 72 hours post-infection (hpi). **b**: Gene clustering based on temporal expression patterns during infection was performed using the Mfuzz R package. The color scale reflects the membership values of the genes; blue, white and pink indicate low membership values, whereas red indicates high membership values. The numbers in brackets indicate the number of genes in each cluster (C) with a membership value ≥ 0.3. **c**: Gene Ontology enrichment analysis revealed that ‘Defense response to virus’ is significantly enriched only in genes of C1 (red bar) but not in those of other clusters (gray bars). The dashed line represents the threshold of the significant enrichment P value with Benjamini-Hochberg (B-H) correction (adjusted p = 0.05). **d**: Heatmap of the activity scores of immune-related pathways at different time points post infection calculated using PROGENy. The color scale indicates the Z score of pathway activity scores, with blue indicating lower activity and red indicating higher activity. **e**: The level of activation (red) or inhibition (blue) of immune-related pathways at 24 and 48 hpi. The regulation level of the pathways is presented as a t-statistic based on PROGENy with linear modeling. The color intensity indicates the -log10(B-H adjusted p values). **f**: Pearson correlation network of immune-related pathways in SARS-CoV-2-infected Calu-3 cells. Nodes represent individual pathways, and the edges between the nodes indicate the correlation of their activity scores. The color scale represents the Pearson correlation coefficient, in which red indicates a positive correlation, and blue indicates a negative correlation. Only edges with an absolute correlation greater than 0.3 are shown. **g**: Western blot analysis showing the dynamic regulation of key members of the canonical NF-κB pathway in N protein-expressing Caco-2 cells infected with SARS-CoV-2 replicon delivery particles (RDPs) lacking the N gene at an MOI of 0.001. The mock infection group was cultured with an equal volume of DMEM. The phosphorylated and total protein levels of p65 and IκBα were detected with the corresponding antibodies, and the signal intensity was quantified using ImageJ. The color scale represents the Z scores of the relative ratios of phosphorylated and total proteins, with red indicating upregulation and blue indicating downregulation. The results are representative of two independent experiments.

To investigate the dynamic regulation of host immune responses, we conducted an RNA-sequencing analysis of SARS-CoV-2-infected Calu-3 cells at a multiplicity of infection (MOI) of 2 at 6, 24, 48 and 72 hours post-infection (hpi). The most significant differences in the transcriptomes were observed between 6 and 24 hpi by principal component analysis (Fig 1a). We next performed a clustering analysis using Mfuzz [36] and categorized the host genes into eight distinct clusters (S1 Table) on the basis of their expression profiles during infection (Fig 1b). Notably, only cluster (C) 1, but not the other clusters, was significantly associated with pathways related to ‘Defense response to virus’ according to enrichment analysis (Figs 1c and S1a). C1 comprises the genes whose expression was upregulated at 24 and 48 hpi but downregulated at 72 hpi, suggesting that the antiviral response is activated upon viral infection while suppressed at later time points. We next performed pathway activity analysis using PROGENy[34] and found the complex regulation of immune-related pathways in host cells during SARS-CoV-2 infection (Fig 1d). Pathways of JAK-STAT, NF-κB and TNF-α showed a pattern similar to that of C1, whose pathway activity scores were upregulated at 24 and 48 hpi while downregulated at 72 hpi (Fig 1d), and the activity of the NF-κB pathway ranked at the top of the 14 immune-related pathways at 24 and 48 hpi (Fig 1e). Consistently, ‘positive regulation of canonical NF-κB signal transduction’ was enriched only for the genes of C1 but not for the other clusters (S1b Fig), indicating that the canonical NF-κB pathway is dynamically regulated by SARS-CoV-2 infection. To examine the regulatory role of the NF-κB pathway, we constructed a correlation network of NF-κB and the other immune-related pathways during SARS-CoV-2 infection and found that NF-κB was positively correlated with the JAK-STAT and TNF-α pathways (Pearson’s correlation coefficient R = 0.88 and 0.96, respectively), indicating a central role of NF-κB in proinflammatory responses (Fig 1f).

To validate this, we measured the levels of key proteins in the canonical NF-κB pathway in N protein-expressing Caco-2 cells (N-Caco-2) infected with SARS-CoV-2 replicon delivery particles (RDPs) constructed based on the Wuhan-Hu-1 strain (NC_045512) lacking the N gene (ΔN SARS-CoV-2 RDPs) [37,38]. We observed that the expression of the active form of the NF-κB transcription factor (TF), phosphorylated p65 (p-p65), was mildly upregulated at 24 hpi compared with that in the mock infection control, peaked at 48 hpi and then decreased at 72 hpi (Fig 1g), consistent with the results of the transcriptomic analysis. To investigate the activation of the canonical NF-κB pathway in more detail, we also analyzed the levels of TAK1 and IκBα. TAK1 activates the IKK complex and leads to phosphorylation and degradation of the inhibitor IκBα, thus releasing NF-κB p50/p65. We found that the level of IκBα phosphorylation (p-IκBα/IκBα) dramatically decreased at 48 and 72 hpi, and similarly, the level of TAK1 diminished gradually during the infection (Fig 1g). In short, SARS-CoV-2 infection leads to dynamic regulation of the canonical NF-κB pathway in the infected cells.

### An integrated prediction model identifies a novel NF-κB regulator

**Fig 2:**
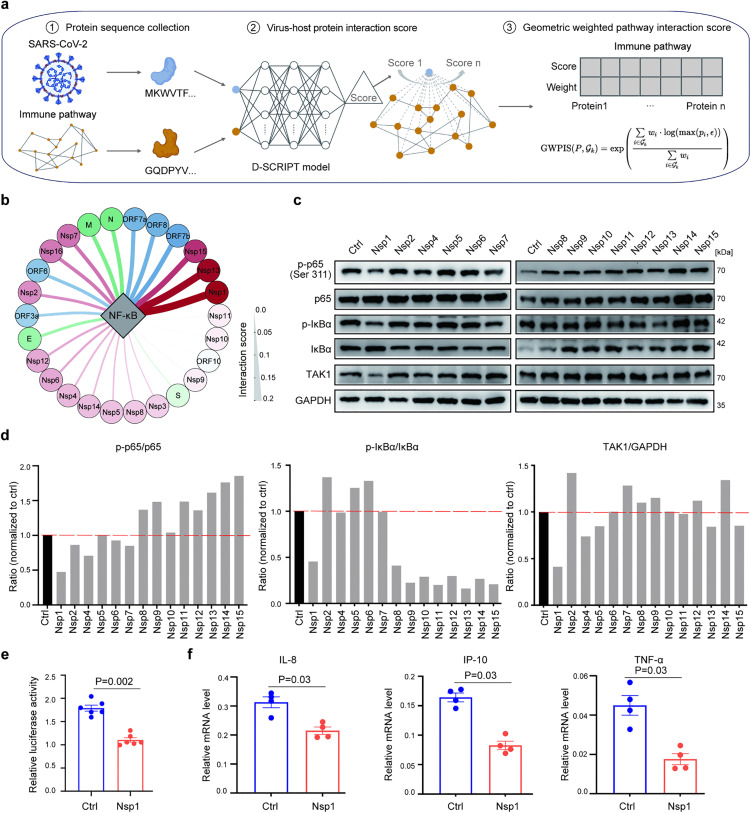
A pretrained protein language model identifies viral protein interactions with the canonical NF-κB pathway. **a**: Schematic overview of the geometric weighted pathway interaction score (GWPIS) for quantifying the interactions between viral proteins and immune-related pathways in infected host cells. Step 1: Data collection of the sequences of SARS-CoV-2 proteins and host proteins in the immune pathways shown in Fig 1d. Step 2: V-H protein interaction score: The D-SCRIPT protein language model was used to compute the interaction scores between SARS-CoV-2 proteins and host proteins in immune-related pathways. Step 3: Pathway-level interaction scores were calculated by integrating the pairwise interaction scores from Step 2 and the gene weights obtained from the PROGENy gene-pathway weight matrix. Created in BioRender. Yang, Q. (2026) https://BioRender.com/41xkykd. **b**: Interactions between SARS-CoV-2 proteins and the canonical NF-κB pathway, as calculated by GWPIS. Each circle represents a SARS-CoV-2 protein, and the color intensity and thickness of the lines reflect the interaction scores. Green, structural proteins (S, E, M, and N); blue, ORF proteins; red/pink, nonstructural proteins. **c**: Western blot analysis showing the regulation of key proteins in the canonical NF-κB pathway in HEK293T cells expressing SARS-CoV-2 Nsps or GFP as a control (Ctrl). The phosphorylated and total protein levels of p65 and IκBα were probed with the corresponding antibodies, and the relative intensity signals were quantified using ImageJ. **d**: Bar chart showing the ratios of phosphorylated and total protein levels in **(c)**. The red dashed line represents the ratio in the GFP-expressing control cells (black). **e**: Activities of the promoter containing NF-κB response elements (κB sites) were measured by a dual luciferase assay. HEK293T cells were transfected with a plasmid expressing a Renilla luciferase reporter driven by a promoter containing 2 κB sites and a control Firefly luciferase 24 hr before transfection of Nsp1 or control GFP-expressing plasmids. The cells were collected 24 h later for the dual luciferase assay. Each dot represents a replicate, and the mean and SEM are shown. P values, Mann-Whitney test. **f**: Relative mRNA levels of *IL-8, IP-10* and *TNF-α* in HEK293T cells transfected with plasmids expressing cMYC-tagged Nsp1 or the GFP control (Ctrl). GAPDH was used as an internal control. Each dot represents a replicate, and the mean and SEM are shown. P values, Mann-Whitney test. The results in **e-f** are representative of two independent experiments.

To elucidate which SARS-CoV-2 proteins are involved in regulating immune responses in infected cells, we developed the geometric weighted pathway interaction score (GWPIS), a novel algorithm to estimate the impact of viral proteins on host immune-related pathways. This approach leverages the sequence representations learned by a pretrained protein language model, D-script [33], and integrates gene weight matrices of the pathway activities derived from PROGENy[34], enabling estimation of pathway-level perturbations driven by the viral proteins (Fig 2a).

We included 26 SARS-CoV-2 proteins (NC_045512.2), comprising 16 nonstructural (Nsps), 4 structural, and 6 accessory proteins (ORFs), and genes of the 14 immune-related pathways regulated by the virus, shown in Fig 1d and 1e for the analysis. The protein-protein interaction scores were calculated by D-script [33] (S2 Table), and the gene weights in each immune-related pathway were determined using PROGENy[34] (S3 Table). Previously, we showed that may play a central role in proinflammatory responses (Fig 1f), and the NF-κB pathway was then used to benchmark GWPIS, which is based on weighted geometric mean (WGM), comparing with 2 commonly used scoring methods: arithmetic mean (AM) and weighted arithmetic mean (WAM) (S2a-S2c Fig). WGM significantly outperformed the other 2 methods in ranking the positive genes, with reported roles in regulating the NF-κB pathway (S2d Fig and S4 Table).

GWPIS values were subsequently calculated for the viral protein-host pathway pairs (S5 Table). The top predicted SARS-CoV-2 immunoregulators were Nsp1, Nsp13, ORF6 and ORF7b, whose GWPIS values were in the top 10%. With respect to the NF-κB pathway, the top three viral candidates were Nsp1, Nsp13 and Nsp15 (Fig 2b). Nsp13 was reported to inhibit NF-κB activation by limiting both p65 phosphorylation and nuclear translocation, thereby dampening downstream immune responses [24]. In contrast, Nsp15 enhances NF-κB activity, particularly under TNF-α stimulation [20]. Notably, the top candidate, Nsp1, with the highest GWPIS value, was not reported to play a role in NF-κB regulation. To validate the results of GWPIS, we conducted an *in vitro* screening assay by transfecting HEK293T cells with plasmids expressing individual SARS-CoV-2 Nsp or GFP as a control and measured the phosphorylation levels of p65 and IκBα, as well as the level of the NF-κB upstream regulator TAK1 (Fig 2c). We found that Nsp15 was the top candidate that upregulated the phosphorylation level of p65, whereas Nsp1 was the top candidate that downregulated the p65 phosphorylation level to 42% of the control level (Fig 2d). Concurrently, Nsp1 also exerted the greatest inhibitory effect on TAK1, reducing the relative expression level to 40% of that of the control (Fig 2d). Using a dual luciferase assay, we confirmed that Nsp1 overexpression inhibited the activation of a promoter containing 2 NF-κB response elements (Fig 2e) and that the transcription levels of proinflammatory factors downstream of the NF-κB pathway, including *IL-8*, *IP-10*, and *TNF-α*, were downregulated in response to Nsp1 overexpression (Fig 2f).

### Nsp1 binds to the TAB1-binding domain of TAK1 and downregulates its protein level

**Fig 3:**
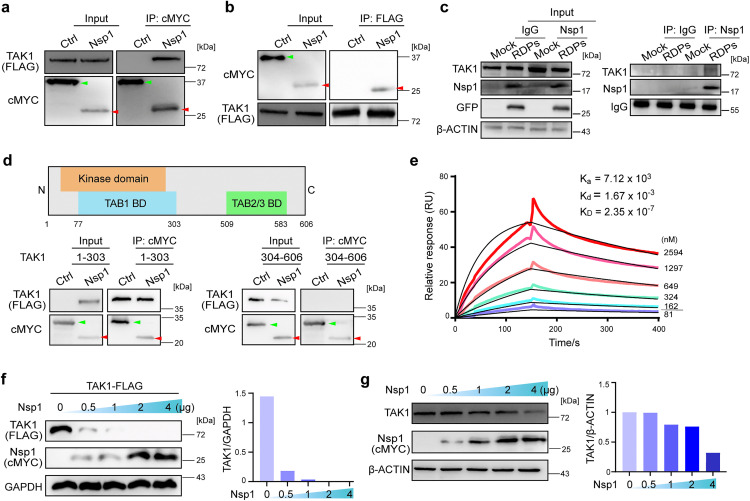
SARS-CoV-2 Nsp1 interacts with the TAK1/TAB1 complex. **a**-**b**: Co-IP and reciprocal co-IP analyses confirm the interaction between Nsp1 and TAK1. HEK293T cells were transfected with plasmids expressing FLAG-tagged TAK1 and cMYC-tagged Nsp1 or GFP as a control (Ctrl). Immunoprecipitation (IP) was performed using anti-cMYC (**a**) and anti-FLAG (**b**) magnetic beads. Nsp1 (red arrowhead) and GFP (green arrowhead) were detected by anti-cMYC, and TAK1 was detected by anti-FLAG antibodies in **a, b** and **f**. **c**: N-Caco-2 cells were infected with ΔN SARS-CoV-2 RDPs and harvested at 48 hpi. IP was performed using anti-Nsp1 antibody or anti-IgG as a control. TAK1 was detected by an anti-TAK1 antibody. **d**: Schematics of the TAK1 truncation proteins (upper panel) and co-IP analysis showing that the N-terminus of TAK1 interacts with Nsp1 (lower panel). HEK293T cells were transfected with plasmids expressing FLAG-tagged N (1-303 aa)- or C (304-606 aa)-terminal truncation of TAK1 24 hr prior to the transfection of cMYC-tagged Nsp1- or GFP (Ctrl)-expressing plasmids. Immunoprecipitation (IP) was performed using anti-cMYC magnetic beads. **e**: SPR (surface plasmon resonance) analysis. Recombinant TAK1 protein (1-303 aa) was diluted and immobilized onto the sensor chip CM5. The recombinant Nsp1 protein was diluted in PBS at different concentrations. The KD value of the Nsp1-TAK1 interaction was 2.3 × 10^-7^ M. **f-g**: HEK293T cells were cotransfected with plasmids expressing FLAG-tagged TAK1 and cMYC-tagged Nsp1 at different concentrations (0.5, 1, 2 or 4 μg) (**f**) or with only the Nsp1-expressing plasmid **(g)**. TAK1 was detected by anti-FLAG in (**f**) or by anti-TAK1 antibody in **(g)**. The relative signals were quantified using ImageJ. The results in **a**-**g** are representative of two independent experiments.

To investigate how Nsp1 attenuates the NF-κB pathway, we transfected HEK293T cells with a plasmid expressing FLAG-tagged Nsp1 protein and performed an immunoprecipitation-mass spectrometry (IP-MS) analysis using an anti-FLAG antibody. Both TAK1 and TAB1 were identified as candidates that interact with Nsp1 (S3a and S3b Figs and S7 Table). As mentioned before, TAK1 is an activator of the canonical NF-κB pathway. TAK1 and TAK1-binding proteins (TAB1, TAB2 and TAB3) form a complex, and the TAK1-TABs complex phosphorylates and activates the IKK complex [39]. The interaction between Nsp1 and TAK1/TAB1 was confirmed by coimmunoprecipitation (co-IP) and reciprocal co-IP assays using HEK293T cells overexpressing Nsp1 and TAK1 or TAB1 protein (Figs 3a, 3b and S3c). Given that the Nsp1 expression level in SARS-CoV-2-infected cells was relatively low [40], we performed a co-IP experiment using large quantities of RDP-infected cells and confirmed the interaction between Nsp1 and TAK1 in N-Caco-2 cells infected with ΔN SARS-CoV-2 RDPs (Fig 3c).

We next investigated which domain of TAK1 interacts with Nsp1 and revealed that Nsp1 binds to the N-terminus (1–303 aa) of TAK1, which comprises the TAB1-binding and kinase domain, but not the C-terminal TAB2/3-binding domain (Fig 3d). Surface plasmon resonance (SPR) analysis using the recombinant Nsp1 and truncated (1–303 aa) TAK1 proteins confirmed the interaction between Nsp1 and the N-terminus of TAK1, with a KD value of 2.3 × 10^-7^ M (Fig 3e). Interestingly, when we cotransfected HEK293T cells with plasmids expressing FLAG-tagged TAK1 (TAK1-FLAG) and Nsp1, the level of overexpressed TAK1-FLAG protein drastically decreased as the level of Nsp1 protein increased (Fig 3f). To test whether Nsp1 also decreases the endogenous TAK1 protein level, we transfected cells with only the Nsp1-expressing plasmid, measured the level of TAK1 using an anti-TAK1 antibody and demonstrated a heightened downregulation of TAK1 as Nsp1 increased (Fig 3g).

Taken together, these data suggest that Nsp1 binds to the N-terminus of TAK1, which contains a TAB1-binding/kinase domain, and decreases the level of the TAK1 protein.

### Interaction between Nsp1 and TAK1 leads to inhibition of the NF-κB, MAPK and AP1 pathways even under proinflammatory stimulation

**Fig 4:**
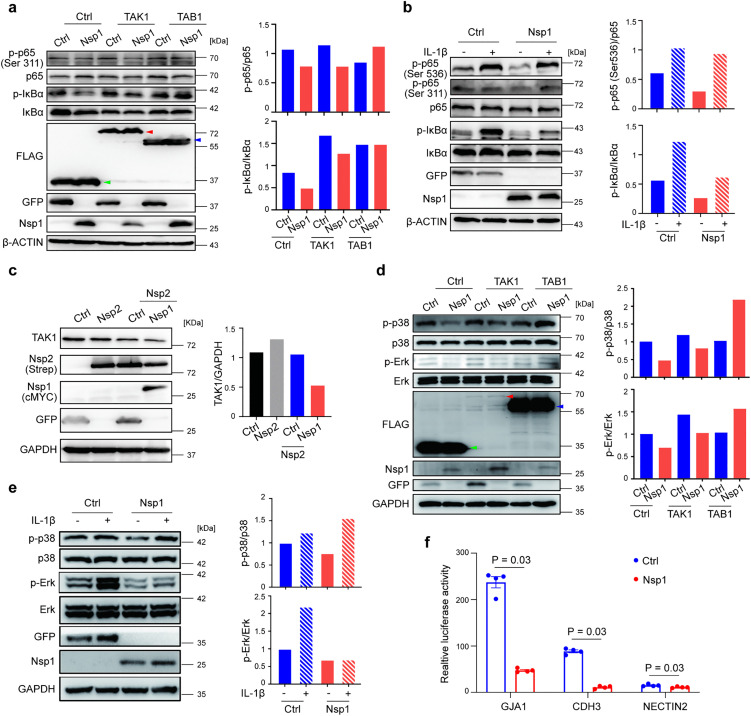
SARS-CoV-2 Nsp1 inhibits TAK1-mediated NF-κB and MAPK signaling. **a**, **d**: Western blot analysis showing the phosphorylated and total protein levels of p65 and IκBα (**a**) or p38 and Erk (**d**) in Nsp1-expressing cells. HEK293T cells were transfected with a plasmid expressing FLAG-tagged GFP (Ctrl), TAK1 or TAB1 24 hr prior to transfection of a plasmid expressing cMYC-tagged Nsp1 (Nsp1-cMYC) or GFP (GFP-cMYC, Ctrl), and the cell lysates were collected 24 hr later. FLAG-tagged GFP (green arrowhead), TAK1 (red arrowhead) and TAB1 (blue arrowhead) were detected by anti-FLAG antibody. GFP was detected by anti-cMYC and anti-Nsp1 antibodies in **a**, **b**, **d** & **e**. The relative intensity signals quantified using ImageJ are shown in the right panel of **a-e**. **b**, **e**: Phosphorylated and total protein levels of p65 and IκBα (**b**) and p38 and Erk (**e**) in Nsp1-expressing cells. HEK293T cells were transfected with the Nsp1-cMYC or GFP-cMYC plasmid for 24 h, after which the culture medium was replaced with DMEM supplemented with 1% FBS for 12 h and then stimulated with IL-1β (20 ng/mL, +) or PBS (-) for 10 min. **c**: HEK293T cells were (co-)transfected with plasmids expressing Strep-tagged Nsp2, Nsp1 (Nsp1-cMYC) and/or GFP (GFP-cMYC, Ctrl). **f**: Activities of the promoters of AP-1 family transcription factors measured by a dual luciferase assay. HEK293T cells were transfected with a plasmid expressing a Renilla luciferase reporter driven by the promoter of *GJA1*, *CDH3* or *NECTIN2* and a control firefly luciferase; 24 h later, the cells were transfected again with an Nsp1- or GFP (Ctrl)-expressing plasmid and collected for the dual luciferase assay 24 h after the second transfection. Each dot represents a replicate, and the mean and SEM are shown. P values, Mann-Whitney test. The results in **a-f** are representative of two independent experiments.

We found that the phosphorylation levels of key molecules in the TAK1 downstream pathways, including p65, IκBα (NF-κB pathway), p38 and ERK (MAPK pathway), were inhibited in Nsp1-expressing HEK293T (Fig 4a and 4d) and A549 cells (S4a and S4b Fig). We next investigated how the interaction between Nsp1 and the TAK1-TAB1 complex affects the downstream signaling. Given that Nsp1 overexpression inhibited the level of TAK1, we transfected the cells with a plasmid expressing FLAG-tagged TAK1 or TAB1 24 hr prior to transfection with a plasmid expressing Nsp1 or GFP control. Interestingly, the phosphorylation levels of p65 and IκBα were both inhibited by Nsp1 even under TAK1 overexpression, with the p-p65/p65 ratio decreased by 32% and the p-IκBα/IκBα ratio downregulated by 24% compared with those in the control (Fig 4a). In contrast, TAB1 overexpression fully compensated for the inhibitory effect of Nsp1 (Fig 4a), presumably due to binding competition between TAB1 and Nsp1 at the TAB1-binding domain of TAK1. We next tested whether the inhibition of p-p65 and p-IκBα remained under external proinflammatory stimulation. When the cells were treated with IL-1β for 10 min, Nsp1 still inhibited the phosphorylation of p65 and Iκbα (Fig 4b), as the phosphorylation of p65 and IκBα decreased by 9% and 49%, respectively, in the Nsp1-expressing cells under IL-1β stimulation.

It was reported that the Nsp2, Nsp6 and N proteins of SARS-CoV-2 interact with TAK1, leading to activation of the NF-κB pathway [21,41]. We therefore tested whether Nsp1 still downregulated the level of TAK1 and its downstream signaling in the presence of Nsp2, Nsp6 and N. We found that although Nsp2 overexpression upregulated the level of TAK1 by 21%, this upregulation was readily counteracted by Nsp1, with the level of TAK1 reduced by 50% compared with that in the control in the presence of Nsp2 (Fig 4c). Similarly, Nsp1 inhibited the level of TAK1 by 77% and 34% in the presence of Nsp6 and N, respectively (S4c and S4d Fig).

The p-p38/p38 and p-ERK/ERK ratios in the MAPK pathway were also inhibited to 48% and 91% of those in the control, respectively, in the Nsp1-expressing cells (Fig 4d). Similarly, the inhibition of p38 and ERK phosphorylation by Nsp1 was maintained under TAK1 overexpression (66% and 54% of the control, respectively), whereas TAB1 overexpression significantly upregulated the phosphorylation level (Fig 4d). Surprisingly, when we treated the cells with IL-1β, we found that Nsp1, but not p38, reduced the phosphorylation level of ERK to 68% of that of the control (Fig 4e).

Another important pathway regulated by TAK1 is AP-1, which is involved in cellular proliferation, transformation and death [42]. Using the dual luciferase assay, we found that Nsp1 overexpression inhibited the promoter activity of AP-1 family transcription factors, including *GJA1* [43,44], CDH3 [45] and NECTIN2 [46] (Fig 4f). This inhibition of the AP-1 pathway is likely responsible for the cell death induced by Nsp1 [47]. Similar to the observations in the NF-κB and MAPK pathways, TAB1 can compensate the suppression of NECTIN2 promoter activity by Nsp1 but not TAK1 (S4e Fig).

Taken together, these findings suggest that Nsp1 binds to the TAB1-binding domain of TAK1, leading to inhibition of TAK1-TAB1 complex downstream signaling, including that of the NF-κB, MAPK and AP-1 pathways. Even in the presence of viral NF-κB activators or IL-1β, whose expression is known to be elevated during SARS-CoV-2 infection [48,49], the canonical NF-κB signaling pathway is still inhibited by Nsp1.

### Nsp1 promotes K48-linked ubiquitination and degradation of TAK1

**Fig 5:**
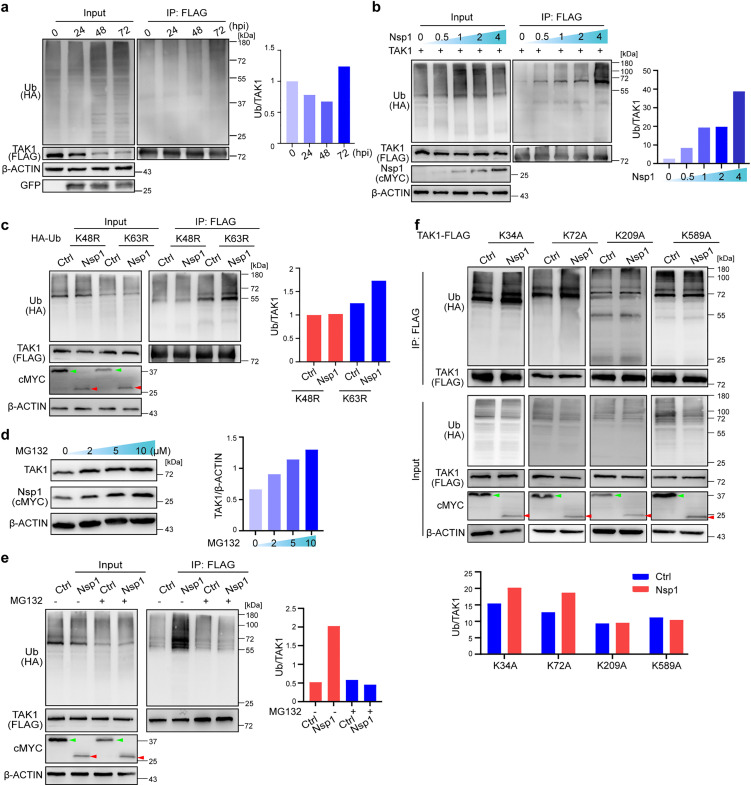
SARS-CoV-2 Nsp1 promotes TAK1 K48-ubiquitin degradation. **a**: The ubiquitination of TAK1 increased in N-Caco-2 cells infected with GFP-expressing ΔN SARS-CoV-2 RDPs at an MOI of 0.01. The cells were transfected with plasmids expressing HA-Ub and FLAG-tagged TAK1 (TAK1-FLAG). Twenty-four hours later, the cells were infected at an MOI of 0.01, and samples were collected at different time points. Ubiquitinated TAK1 was immunoprecipitated (IP) using anti-FLAG magnetic beads, and the ubiquitination level was analyzed with an anti-ubiquitin antibody. TAK1 was detected by anti-FLAG. The relative signals quantified by ImageJ are shown in the right panels of **a-c** and **e-f**. **b**: HEK293T cells were transfected with plasmids expressing HA-Ub and TAK1-FLAG 24 h prior to transfection with different quantities of plasmids expressing cMYC-tagged Nsp1 (Nsp1-cMYC; 0, 0.5, 1, 2 or 4 μg). Nsp1 was detected by an anti-cMYC antibody in **b**-**d**. **c**: HEK293T cells were transfected with plasmids containing TAK1-FLAG and wild-type (WT) or mutant HA-Ub in which the corresponding ubiquitin-accepting lysine (K) residue was replaced with arginine (R), and 24 h later, the cells were transfected again with the cMYC-tagged Nsp1 (Nsp1-cMYC) or GFP control (Ctrl, GFP-cMYC) plasmid. Green arrowhead, GFP-cMYC; red arrowhead, Nsp1-cMYC in **c**, **e** and **f**. **d**: HEK293T cells were transfected with the Nsp1-cMYC plasmid 24 hr prior to treatment with different concentrations of MG132 (0, 2, 5 or 10 μM) for 12 hr. The relative signals between TAK1 (by anti-TAK1) and β-ACTIN (by anti-β-ACTIN) were quantified by ImageJ and are shown in the right panel. **e**: HEK293T cells were treated with 10 μM MG132 or DMSO (Ctrl) prior to transfection with the TAK1-FLAG and HA-Ub plasmids, and 24 h later, they were transfected again with the Nsp1-cMYC or control GFP-cMYC (Ctrl) plasmid. **f**: Polyubiquitination levels of TAK1 mutants in which the lysine (K) residue at position 34, 72, 209 or 589 was mutated to alanine (A). HEK293T cells were first transfected with plasmids expressing HA-Ub and wild-type (WT) or mutant TAK1-FLAG; 24 h later, the cells were transfected again with Nsp1-cMYC or GFP-cMYC (Ctrl) plasmids. The results in **a**, **c**, **d**, **e**, and **f** are representative of two independent experiments.

Previously, we demonstrated that the level of TAK1 was significantly reduced in N-Caco-2 cells infected with ΔN SARS-CoV-2 RDPs at 72 hpi (Fig 1g). We further analyzed the mRNA level of TAK1 and confirmed a marked downregulation after RDP infection compared with the mock control (S5a Fig). However, the mRNA levels at 24, 48, and 72 hpi did not significantly differ (S5a Fig). These findings suggest that the downregulation of protein levels at 72 hpi may be due to posttranscriptional or posttranslational mechanisms. Given that TAK1 is known to be subjected to ubiquitin-dependent degradation [50,51], we examined whether the downregulation of TAK1 in the RDP-infected cells was due to the increased ubiquitination level. By transfecting a plasmid expressing FLAG-tagged TAK1 protein into the N-Caco-2 cells 24 h prior to infection with the ΔN SARS-CoV-2 RDPs, we analyzed the ubiquitination level of FLAG-tagged TAK1. The ubiquitination of TAK1-FLAG decreased at 48 hpi but sharply increased at 72 hpi (Fig 5a), suggesting that the prominent downregulation of TAK1 protein expression at 72 hpi may be due to the upregulated ubiquitination level of TAK1.

We next investigated whether Nsp1 is involved in the ubiquitination of TAK1. By transfecting HEK293T cells with plasmids expressing FLAG-tagged TAK1 and HA-tagged ubiquitin (HA-Ub) with or without the Nsp1-expressing plasmid, we found that the ubiquitination level of TAK1 significantly increased by 26% in Nsp1-expressing cells compared with that in control cells (S5b Fig), while the ubiquitination level of TAB1 was not affected by Nsp1 (S5c Fig). Importantly, the polyubiquitination level of TAK1 increased as the level of Nsp1 increased (Fig 5b).

We next investigated which types of polyubiquitin chains of TAK1 were promoted by Nsp1 by transfecting cells with HA-tagged wild-type Ub (WT-Ub) or K48R-/ K63R-Ub mutant in which the lysine residues of the ubiquitin were mutated to arginine. The K48R-Ub only allows K63-linked polyubiquitination, whereas the K63R-Ub lacks K63-linked polyubiquitination [52]. We found that the level of TAK1 ubiquitination was increased by 38% in Nsp1-expressing cells transfected with the K63R-Ub plasmid, which retained only K48-linked polyubiquitin, but not in those with K48R-Ub, which lacks the K48 linkage type (Fig 5c), suggesting that Nsp1 significantly promotes the K48-linked polyubiquitin of TAK1 but not the K63 linkage type.

K48-linked ubiquitination is associated with proteasomal protein degradation [53]. These findings suggest that Nsp1 may lead to increased proteasomal degradation of TAK1, thus reduce the level of TAK1. To test this hypothesis, we treated Nsp1-expressing cells with MG132, which inhibits proteasome activity [54], and found that as the concentration of MG132 increased (from 0 to 10 μg), the expression of endogenous TAK1 protein increased (Fig 5d). Furthermore, treatment with MG132 (10 μg) completely reversed the Nsp1-promoted polyubiquitination of TAK1 (Fig 5e). Taken together, these results indicate that Nsp1 promotes K48-linked polyubiquitination of TAK1, targeting it for proteasomal degradation and thereby resulting in a reduced protein level of TAK1.

Compared with K48, K63-linked polyubiquitination of TAK1 is better characterized [55]. Only the lysine residue K72 was reported to accept K48-linked polyubiquitination [56]. We thus analyzed K72, together with 3 lysine residues reported to have major functions, K34, K209 and K589 (annotated in ENST00000369329.8, also known as K562 [39,55,57] in ENST00000369332.7). By transfecting HEK293T cells with plasmids expressing HA-Ub, Nsp1, and different mutant TAK1 (TAK1^K34A^, TAK1^K72A^, TAK1^K209A^ and TAK1^K589A^) in which the corresponding ubiquitin-accepting lysine was mutated to alanine, we found that the polyubiquitination level of TAK1 was not affected by Nsp1 in the TAK1^K209A^ and TAK1^K589A^ mutants, whereas Nsp1-mediated enhancement of polyubiquitination was still observed in the TAK1^K34A^ and TAK1^K72A^ mutants (Fig 5f), suggesting that Nsp1 affects the conjugation of ubiquitin chains at K209 and K589 but not at K34 or K72.

### TRIM21 is an E3 ubiquitin ligase for TAK1 and is involved in the Nsp1-mediated upregulation of TAK1 ubiquitination

**Fig 6:**
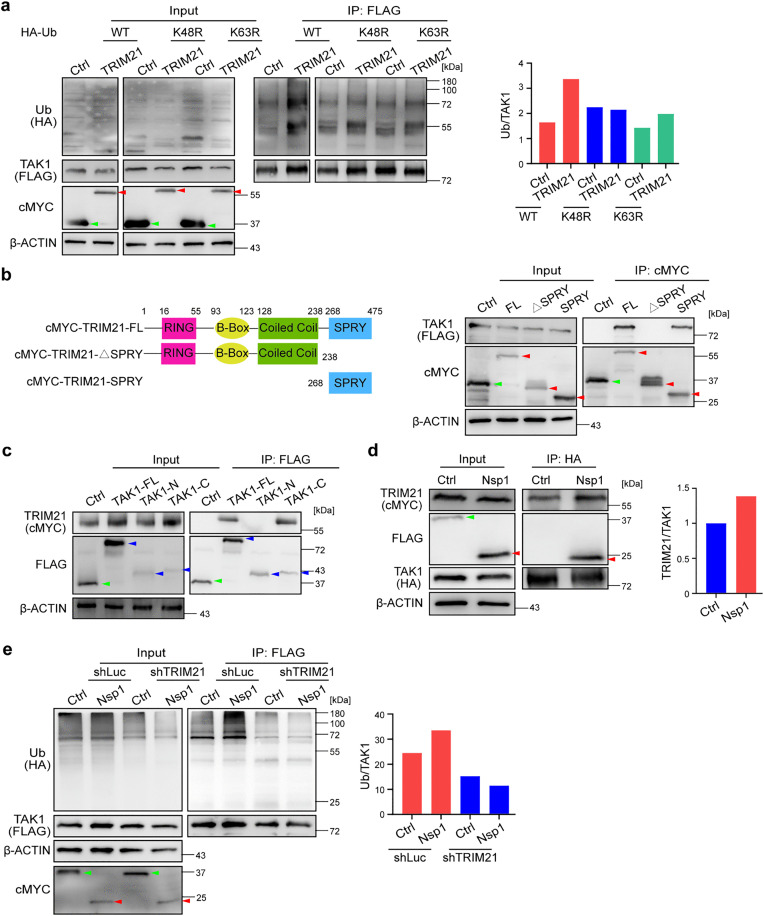
TRIM21 is involved in the Nsp1-mediated upregulation of TAK1 ubiquitination. **a**: HEK293T cells were transfected with plasmids expressing FLAG-tagged TAK1 (TAK1-FLAG) and wild-type (WT) or mutant (K48R/K63R) HA-Ub, and 24 h later, were transfected again with a plasmid expressing cMYC-tagged TRIM21 (cMYC-TRIM21) or GFP (GFP-cMYC, Ctrl). Ubiquitinated TAK1 was immunoprecipitated (IP) by anti-FLAG magnetic beads, and the ubiquitination level was analyzed by an anti-HA antibody. GFP (green arrowhead) and TRIM21 (red arrowhead) were detected by anti-cMYC and anti-TAK1 with anti-FLAG antibodies in **a-b**. The relative signals of HA-Ub and TAK1-FLAG quantified by ImageJ are shown in the right panels in **a** & **e**. **b**: Plasmids expressing cMYC-tagged full-length (FL) or truncated TRIM21 mutants expressing only SPRY or lacking (Δ) the SPRY domain were constructed (left panel). SPRY, SPla and the RYanodine Receptor domain. HEK293T cells were cotransfected with plasmids expressing TAK1-FLAG and FL or truncated cMYC-TRIM21 or GFP. Immunoprecipitation (IP) was performed using anti-cMYC magnetic beads (right panel). **c**: HEK293T cells were cotransfected with plasmids expressing cMYC-TRIM21 and FLAG-tagged full-length (FL) or truncated N-terminal (1-303 aa, N) or C-terminal (304-606 aa, C) TAK1 mutants. FLAG-tagged GFP (green arrowhead) and TAK1 (blue arrowhead) were detected with an anti-FLAG antibody. **d**: HEK293T cells were transfected with plasmids expressing HA-tagged TAK1 and cMYC-TRIM21 24 h later and transfected with a plasmid expressing FLAG-tagged Nsp1 (red arrowheads) or a GFP control (Ctrl, green arrowhead). Immunoprecipitation (IP) was performed using anti-HA magnetic beads. The relative signals of cMYC-TRIM21 and TAK1-HA quantified using ImageJ are shown in the right panel. **e**: HEK293T cells were transfected with a lentiviral vector encoding shRNA targeting TRIM21 (shTRIM21) or luciferase (shLuc) as a control. After puromycin (10 μg/mL) selection for 72 hr, the cells were transfected with an HA-Ub plasmid, and 24 hr later, transfected with an Nsp-cMYC (red arrowheads) or a GFP-cMYC plasmid (Ctrl, green arrowheads). The results in **a**, **d** and **e** are representative of two independent experiments.

We found that TRIM21, a member of the tripartite motif (TRIM) family and a RING finger domain-containing E3 ligase [58], was among the interacting candidates of Nsp1 (S5d and S7 Tables). We therefore tested whether TRIM21 is responsible for TAK1 ubiquitination. By transfecting cells with a plasmid expressing WT, K48R- or K63R-Ub with or without the cMYC-tagged TRIM21 plasmid, we found that the ubiquitination level of TAK1 was upregulated to twice that of the control level in the WT-Ub-expressing cells when TRIM21 was overexpressed (Fig 6a). In contrast, the polyubiquitination level of TAK1 was unaffected in cells expressing K48R-Ub, suggesting that compared with WT-Ub, TRIM21 is capable of promoting the K48-linked polyubiquitination of TAK1 and is likely partially responsible for K63-linked polyubiquitination, as the level of upregulation was reduced in K63R-Ub cells (Fig 6a).

To determine how TRIM21 interacts with TAK1, different truncation mutants of TRIM21 were constructed to perform co-IP assays (Figs 6b and S5e). We found that the mutant lacking the SPRY (SPla and the RYanodine Receptor) domain lost the binding capacity to TAK1. In contrast, the mutant containing only the SPRY domain was able to bind to TAK1, indicating that TRIM21 interacts with TAK1 with the SPRY domain (Figs 6b and S5e). Similarly, TAK1 truncation mutants were used to determine how TAK1 interacts with TRIM21. We found that TRIM21 binds to the C-terminus of TAK1 (Figs 6c and S5f). Importantly, the interaction between TRIM21 and TAK1 increased by 39% in the presence of Nsp1 (Fig 6d).

To further investigate whether TRIM21 is responsible for the Nsp1-mediated upregulation of TAK1 ubiquitination, we analyzed the polyubiquitination level of TAK1 in Nsp1-expressing cells cotransfected with a lentiviral vector encoding shRNA targeting TRIM21 or luciferase as a control (S5g Fig). Notably, TRIM21 knockdown did not affect the TAK1 protein level (S5h Fig); however, shTRIM21 ablated the increased ubiquitination level of TAK1 induced by Nsp1 (Fig 6e). These results collectively indicate that Nsp1 increases the level of TRIM21-mediated TAK1 polyubiquitination.

## Discussion

Significant changes in immune-related pathways during viral infection have been revealed by various transcriptomic analyses [59–61]; however, the specific viral proteins driving this regulation remain unknown. To address this, GWPIS was developed to bridge the gap between protein-protein interactions and pathway-level regulation. By weighting the virus-host protein-protein interactions, viral proteins that target immune-related pathways were prioritized. Nsp1 was predicted and experimentally verified as a key viral protein that regulates the NF-κB pathway, indicating the robustness of GWPIS.

GWPIS was built as a modular framework so that the key components can be adapted to different research settings. D-SCRIPT was used in this study for the interaction module, as it has been applied in SARS-CoV-2 studies [33] and showed good agreement with the experimental data [62]. For other pathogens, this module can be replaced with other pathogen-host interaction predictors tailored to the relevant context, such as DeepViral [29] for influenza, Zika, and dengue viruses or deepHPI [30] for bacterial proteins. However, the limitations of GWPIS should also be acknowledged. As a PPI-based framework, GWPIS depends strongly on the reliability of protein-protein interactions. Meanwhile, our method relies on PROGENy-derived gene weights, which contains only a limited number of immune-related pathways*.*

As a key regulator of inflammation, NF-κB mediates vital antiviral responses; however, excessive activation contributes to infection-induced tissue injury [63,64]. Hyperactivation of the NF-κB pathway contributes to COVID-19 pathogenesis, particularly in severe cases [65]. NF-κB activation drives the transcriptional upregulation of a broad spectrum of molecules, including proinflammatory cytokines (IL-1β, IL-6, TNF-α), chemotactic factors (MIP-1α, RANTES), endothelial adhesion molecules (ICAM-1, VCAM-1), acute-phase reactants (SAA), and enzymes critical to inflammation (iNOS, COX-2) [66–68]. It was reported that suppressing NF-κB activity effectively inhibits viral replication and the release of proinflammatory cytokines [69]. COVID-19 drugs, including paxlovid [70], glucocorticoids (methylprednisolone and dexamethasone) [71], IL-6/IL-6R monoclonal antibodies [72] and the JAK inhibitor baricitinib [73], have been shown to have modulatory effects on NF-κB activation. By inhibiting TNF-α-induced NF-κB activation, TNF-α blockers (e.g., infliximab and adalimumab) have shown the potential of lowering hospitalization risk and COVID-19 severity [74]. Clinical studies indicated that early anakinra administration in patients with moderate-to-severe COVID-19 significantly improved outcomes [75], and as an IL-1α/β inhibitor, anakinra also suppresses NF-κB activation. In addition, pharmacological inhibition of NF-κB using natural compounds [76], traditional medicines [77], or vasoactive intestinal peptide [78] attenuated hyperactivation of the pathway and significantly reduced the COVID-19-associated cytokine storm [69].

However, how SARS-CoV-2 infection regulates the NF-κB pathway remains elusive. Many SARS-CoV-2 proteins, including the S protein [13], M protein [14], Nsp5 [15], Nsp6 [21], Nsp14 [16,17], Nsp15 [20], ORF7a[14,21] and ORF3a[20], were reported to activate the NF-κB pathway. A few viral proteins, including Nsp13 [21], ORF6 [16] and Nsp9 [20], have been reported to inhibit NF-κB signaling. However, the observations of several studies conflicted. For example, the N protein was reported to undergo liquid-liquid phase separation when it binds to viral RNA, recruit TAK1 and the IKK complex and enhance NF-κB signaling [18]. Another study demonstrated that N protein suppresses the activation of the NF-κB pathway by disrupting the assembly of the TAK1-TAB2/3 complex, thereby inhibiting the downstream signal transduction [41]. Moreover, Nsp3 was reported to enhance the phosphorylation of both IκBα and IκBβ by interacting with BRAP, which subsequently promotes the nuclear translocation of the NF-κB subunits p50 and p65 [19]. However, the interaction between NEMO and Nsp3 leads to polyubiquitylation and degradation of NEMO and subsequently disrupts NF-κB activation [22].

**Fig 7:**
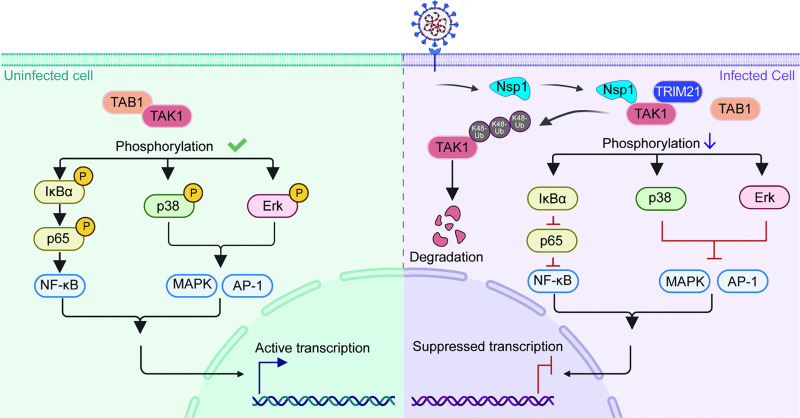
SARS-CoV-2 Nsp1 inhibits TAK1-mediated immune pathways by promoting the TRIM21-dependent K48-linked polyubiquitination of TAK1. In uninfected cells (left), TAB1 binds to TAK1, and the TAK1-TAB1 complex phosphorylates downstream regulators, including IκBα, p38, and Erk, which in turn activate the downstream NF-κB, MAPK and AP-1 pathways. In SARS-CoV-2-infected cells (right), Nsp1 binds to TAK1 at the N-terminal TAB1-binding domain, preventing the formation of the TAK1-TAB1 complex and promoting the binding of TRIM21 to the C-terminus of TAK1. As a result, Nsp1 promotes the proteasomal degradation of TAK1 by enhancing the TRIM21-dependent K48-linked polyubiquitination (Ub) of TAK1, thereby attenuating the activity of the NF-κB and AP-1 pathways. Created in BioRender. Yang, Q. (2026) https://BioRender.com/41xkykd.

In this study, we revealed that SARS-CoV-2 dynamically modulates NF-κB activity during infection, which was activated at 24 and 48 hpi but suppressed at 72 hpi. We uncovered a central role of Nsp1 in the inhibition of the NF-κB pathway, which was achieved by blocking the formation of the TAK1-TAB1 complex, as both Nsp1 and TAB1 bind to TAK1 at the TAB1-binding/kinase domain. Nsp1-mediated TAK1 suppression is potent and counteracts the activation effect of other viral proteins, including N, Nsp6 and Nsp2, as well as proinflammatory cytokines, such as IL-1β. Nsp1 decreases the level of TAK1 by promoting TRIM21-dependent K48-linked polyubiquitination and subsequent proteasomal degradation (Fig 7). As a key kinase involved in regulating the NF-κB, MAPK and AP-1 signaling pathways [39,79], the degradation of TAK1 leads to the downregulation of p-p65, p-IκBα, p-p38 and p-ERK and the inhibition of AP-1 family transcription factors. As a result, the levels of proinflammatory factors such as *IL-8, IP-10 and TNF-α* were reduced, and cell death was promoted at the late stage of infection.

Ubiquitination has emerged as a critical mechanism exploited by viruses to manipulate host factors essential for infection, including proteasomal degradation, immune signaling and viral restriction [80–82]. During SARS-CoV-2 infection, the ubiquitin system is exploited by the virus to suppress the antiviral defenses of the host. Nsp6 impairs TRIM4-mediated MDA5 K63-linked polyubiquitination and thereby downregulates the expression of IFN-I [83]. ORF7b suppresses the antiviral signaling pathway by preventing K63-linked polyubiquitination of MAVS and adaptor recruitment [84]. Unlike K63-linked ubiquitin chains, which play important roles in immune signaling [85], K48-mediated ubiquitin linkages target proteins for proteasomal degradation [86–88] and are also hijacked by viruses. The influenza virus PB2 protein directly binds to JAK1, inducing K48 polyubiquitination at the K859/K860 site, which triggers proteasome-dependent JAK depletion to facilitate viral transcription and replication [89].

As a key immunoregulator, TAK1 is comprehensively regulated by the ubiquitin system [55], including by K63-linked ubiquitination by TRAF6 [42,57,90,91], TRAF2 [42], TRIM8 [92,93], CHIP [94] and Sef-S [95] and K48 ubiquitination by TRIM13 [96], TRIM16 [97], ITCH [56] and FBXW2 [98]. In this study, we identified a novel E3 ligase for TAK1, TRIM21, which interacts with the C-terminus of TAK1 through the SPRY domain and promotes K48-linked polyubiquitination of TAK1. This posttranslational modification is exploited by SARS-CoV-2 via Nsp1, which inhibits the formation of the TAK1-TAB1 complex but promotes the TAK1-TRIM21 interaction, leading to the downregulation of TAK1.

Here, we propose a key regulatory role of SARS-CoV-2 Nsp1 in the TAK1-mediated signaling pathways by promoting TRIM21-dependent K48-linked ubiquitination of TAK1 and targeting TAK1 for proteasomal protein degradation. Nsp1-induced TAK1 degradation is critical for inhibiting the NF-κB response, highlighting the sophisticated viral mechanisms of immune evasion. A comprehensive understanding of the modulation of the host NF-κB signaling pathway by SARS-CoV-2 will provide critical theoretical foundations for the development of NF-κB-targeted drugs or immunotherapies.

## Method details

### Cell lines and cell culture

The HEK293T, A549 and Calu-3 cell lines were originally obtained from the ATCC. HEK293T and A549 cells were maintained in DMEM (HyClone, #SH30022.01) supplemented with 10% fetal bovine serum (FBS, Cell-Box, #SV30087.03) and 1% penicillin-streptomycin (Gibco, #15140122). Calu-3 cells were maintained in Eagle’s minimal essential medium (EMEM; Lonza, #BE12-136F), supplemented with 8% FBS (Thermo Fisher, #A3382001), 2 mM L-glutamine (Sigma-Aldrich, #G7513), 100 IU/mL penicillin and 100 µg/mL penicillin (Sigma-Aldrich, #P4458), 1 × nonessential amino acids (NEAA; Lonza, #BE13-114E), and 1 mM sodium pyruvate (Thermo Fisher, #11360039).

A Caco-2 cell line stably expressing the SARS-CoV-2 nucleocapsid protein (N-Caco-2) was used to establish SARS-CoV-2 replicon delivery particles (RDPs) constructed from the Wuhan-Hu-1 strain (NC_045512) lacking the N gene (ΔN SARS-CoV-2 RDPs) [37]. N-Caco-2 cells were cultured in DMEM (HyClone, #SH30022.01) supplemented with 10% FBS (Gibco, #10270–106), 1% penicillin-streptomycin (10,000 U/mL) (Gibco, #15140122) and 2 μg/mL puromycin (Beyotime, #ST551). All cell cultures were maintained at 37°C in an atmosphere of 5% CO_2_ and 95% to 99% humidity. All the cell lines were routinely tested for mycoplasma contamination.

### Culture of SARS-CoV-2 and ΔN-SARS-CoV-2 RDPs

Calu-3 cells were infected with SARS-CoV-2 (BetaCoV/Wuhan/IVDC-HB-01/2019 strain) at an MOI of 2 for 1 h at 37°C with shaking. After the inoculum was removed, the cells were washed twice with PBS and maintained in EMEM supplemented with 2% FCS, L-glutamine, NEAA and antibiotics. The supernatants and cells were collected at 6, 24, 48 and 72 hpi for RNA extraction. Experiments involving the infectious SARS-CoV-2 virus were performed in a designated biosafety level 3 (BSL-3) laboratory in accordance with institutional guidelines at Leiden University Medical Center (LUMC).

N-Caco-2 cells were infected with ΔN-SARS-CoV-2 RDPs for 24 hr at 37°C. After the inoculum was removed, the cells were washed twice with PBS and maintained in DMEM supplemented with 10% FBS and 1% penicillin-streptomycin (10,000 U/mL). Experiments involving SARS-CoV-2 RDPs were performed in a Biosafety Level 2 (BSL-2) laboratory at West China Hospital of Sichuan University (WCHSCU) in accordance with national and institutional guidelines.

### DNA constructs and cell transfection

The SARS-CoV-2 Nsps-expressing plasmids [99] were kindly provided by Prof. Kefeng Lu (Sichuan University, China). All genes were codon optimized with the exception of Nsp3 and Nsp16, and cloned into the mammalian expression vector pLVX-EF1alpha with a 2 × Strep tag [99]. In addition, the codon-optimized Nsp1 was cloned into pcDNA3.1 in frame with a C-terminal FLAG or cMYC tag. The coding sequences of TAK1 and TAB1 were amplified from the cDNA of HEK293T cells and cloned into pcDNA3.1 in frame with a C-terminal FLAG tag. The truncations of TAK1 were generated by subcloning the fragments of the coding sequences into pcDNA3.1 in frame with the C-terminal FLAG tag. TAK1 mutants with lysine residues mutated to alanine (K34RA, K72A, K209A and K589A) were constructed via PCR-based site-directed mutagenesis. The coding sequences of TRIM21 or truncated mutants were amplified from the cDNA of HEK293T cells and subcloned into pcDNA3.1 with an N-terminal cMYC tag. For the luciferase reporter assay, the promoter sequences of *GJA1, CDH3 and NECTIN2* were amplified from the DNA of HEK293T cells and cloned into luciferase plasmids based on the pGL4.7 vector (Promega, #E6881) as described previously [100]. A promoter sequence containing two NF-κB response elements (5’-GGGRNNNYCC-3’) [101] was synthesized, cloned into the dual luciferase plasmid. The sequences of the primers and oligos used in this study are listed in S6 Table. The constructed plasmids were transfected into cells using LipoMax Transfection Reagent (Sudgen, #32012) according to the manufacturer’s instructions.

### Immunoprecipitation and immunoblotting

#### Protein extracts.

For immunoblotting, the cells were lysed with RIPA lysis buffer (Thermo Fisher Scientific, #89900) supplemented with 10 mM EDTA-free Protease Inhibitor Mini Tablets (Thermo Fischer Scientific, #A32955) and incubated on ice for 30 min. After centrifugation, the protein content in the supernatant was quantified using a Pierce BCA Protein Assay Kit (Thermo Fisher Scientific, #23227).

#### Immunoprecipitation.

Cells were lysed with cell lysis buffer (Cell Signaling Technology, #9803) supplemented with 10 mM protease inhibitor mini tablets and incubated overnight with anti-DYKDDDDK magnetic beads (Thermo Fisher Scientific, #A36798) or anti-cMYC magnetic beads (Thermo Fisher Scientific, #88842) according to the manufacturer’s instructions. The beads were then washed five times in wash buffer containing 50 mM Tris-HCl (pH 8.0), 1% NP-40 (v/v), 150 mM NaCl, 2 mM EDTA (pH 8.0) and 0.5% sodium deoxycholate, and the immunoprecipitates were subsequently eluted with 1 × SDS loading buffer (Sangon Biotech, #C508320).

#### Mass spectrometry.

The immunoprecipitates were loaded onto separate lanes of a 10% SDS polyacrylamide gel and electrophoresed for 20 min. The entire gel region containing the proteins was cut and subjected to in-gel trypsin digestion and subsequent recovery of the peptides as described previously [102]. Q Exactive Plus (Thermo Fisher Scientific), coupled online to the UPLC, was used for the proteomics analysis. The MS data were processed using Proteome Discoverer 2.1. Tandem mass spectra were searched against the UniProt database. A list of the identified candidates is provided in S7 Table.

#### Immunoblotting.

Protein extracts or immunoprecipitates were separated by 10% SDS polyacrylamide gel electrophoresis (PAGE) and transferred to polyvinylidene difluoride membranes (Millipore, #ISEQ00010). The blots were probed with primary antibodies in universal antibody diluent (NCM Biotech, #WB500D) for 1 h, followed by three washes with Tris-buffered saline supplemented with Tween 20 (Sigma, #V900548) and then incubation with HRP-conjugated goat anti-rabbit antibodies (HuaBio, #HA1001) or HRP-conjugated goat anti-mouse antibodies (HUABIO, #HA1006). The chemiluminescence signal was detected using a ChemiDOCTMMP Imaging System (Bio-Rad Laboratories).

The following primary antibodies were used in this study: anti-SARS-CoV-2 NSP1 (ABclonal, #A20200), anti-TAB1 (CST, #3226), anti-TAK1 (CST, #5206S), anti-phospho-IκBα-S32 (ABclonal, #AP0707), anti-phospho-Erk1 (T202 + Y204) + Erk2 (T185 + Y187) (HUABIO, #ET1610–13), anti-phospho-p38 MAPK (Thr180/Tyr182) (CST, #9211), anti-p-RELA/NF-κB p65 (Ser311) (Santa Cruz Biotechnology, #sc-135769), anti-phospho-NF-kappaB p65 (Ser536) (CST, #3033T), anti-IκBα (ABclonal, #A19714), anti-Erk1/2 (HUABIO, #ET1601–29), anti-p38 MAPK (CST, #9212), anti-RELA/NF-κB p65 (Santa Cruz Biotechnology, #sc-514451), anti-Ubiquitin N-terminal (PTM Biolabs, #PTM-1106RM), anti-GFP (Proteintech, #66002), anti-HA tag (ABclonal, #AE008), anti-DYKDDDDK (FLAG) (CST, #14793), anti-Strep (HUABIO, #HA722229), anti-GAPDH (CST, #2118) and anti-cMYC (Sigma, #C3956).

### RNA interference

Lentiviral vectors expressing shRNA targeting TRIM21 or luciferase (Luc) as a control were constructed by cloning shRNAs into pLKO.1-TRC (Addgene, #10878). The pLKO.1-TRC, psPAX2 (Addgene, #12260) and pMD2. G (Addgene, #12259) were cotransfected into HEK293T cells with LipoMax Transfection Reagent (Sudgen, #32012) according to the manufacturer’s instructions. The medium was replaced with fresh DMEM containing 10% FBS and 1% penicillin/streptomycin 12 h posttransfection. The culture medium was harvested by pelleting the cells at 500 × g for 5 min at 48 hr post-transfection. HEK293T cells were transduced with lentivirus expressing shRNAs by incubation with 1 μg/mL hexadimethrine bromide (polybrene) for 48 hr, and selection was performed with puromycin (10 μg/mL) for 3 days. The knockdown efficiency was validated using RT-qPCR. The sequences of the shRNAs and the primers used are listed in S6 Table.

### RNA extraction and RT-qPCR

Total RNA was isolated from the cells using TRIzol reagent (Invitrogen, #15596018) in accordance with the manufacturer’s instructions. Complementary DNA (cDNA) was obtained using a HiScript III All-in-one RT SuperMix Perfect For qPCR kit (Vazyme, #R333-01) according to the manufacturer’s protocol. Quantitative PCR (qPCR) was performed using SYBR Green Master Mix (Yeasen, #11184ES08) on a CFX96 Touch Real-Time PCR Detection System (Bio-Rad). The sequences of the primers are listed in S6 Table.

### Luciferase reporter assays

To determine gene expression, we constructed dual-luciferase plasmids based on the pGL4.7 vector (Promega, #E6881). *GJA1*, *CDH3,* and *NECTIN2* promoters or a promoter with a 2 NF-κB response element sequence were cloned and inserted into luciferase reporter plasmids. HEK293T cells (5 × 10^5^) were seeded in 24-well plates and transfected with the reporter plasmid; 24 h later, a plasmid expressing Nsp1 or GFP was transfected. The cells were pelleted and collected 24 h after the second transfection. Luciferase assays were performed according to the manufacturer’s instructions (Yeasen, #11401ES60) and measured on an InfiniteM200 (Tecan). The promoter activity was determined as the ratio of Renilla luciferase activity under the control of the *GJA1*, *CDH3,* or *NECTIN2* promoters or a promoter with 2 NF-κB response elements and an internal control of firefly luciferase.

### Ubiquitination assays

Plasmids expressing HA-tagged wild-type or mutant ubiquitin (WT-Ub, K48R-Ub or K63R-Ub) were kindly provided by Prof. Da Jia (Sichuan University, China). HEK293T cells were transfected with a plasmid expressing WT-Ub, K48R-Ub or K63R together with a plasmid expressing FLAG-tagged TAB1 or WT/mutant TAK1; 24 h later, the cells were again transfected with a plasmid expressing cMYC-tagged Nsp1 or GFP control. Plasmid-transfected cells were collected at 24 h post secondary transfection, lysed and subjected to immunoprecipitation using anti-DYKDDDDK magnetic beads (Thermo Fisher Scientific, #A36798) as described above.

N-Caco-2 cells were first transfected with plasmids expressing HA-tagged ubiquitin (HA-Ub) and FLAG-tagged TAK1 (TAK1-FLAG). Twenty-four hours later, the cells were infected with GFP-expressing ΔN SARS-CoV-2 RDPs at an MOI of 0.01, and samples were collected at different time points. Ubiquitinated TAK1 was immunoprecipitated (IP) using anti-DYKDDDDK magnetic beads (Thermo Fisher Scientific, #A36798) as described above. The ubiquitination level was analyzed by an anti-ubiquitin antibody.

### Expression of the Nsp1 recombinant protein

An 8 × His tag was fused to the N-terminus of the Nsp1 protein, and the encoded sequence was cloned into the pTO-T7 plasmid, which was expressed in *E. coli* ER2566 (NEB, #E4130G). The cells were cultured at 37°C for 3.5 hr and subsequently at 28°C for 10 hr. Afterward, PBS was added at a ratio of 15 mL per gram of cell pellet, and the cells were lysed by sonication. The supernatant from the lysate was purified using an AKTA purification system (ӒKTA pure, Cytiva) with Ni-Excel resin (Cytiva, #17-3712-03). The impurities were removed with 3 mM imidazole, and the target protein was eluted with 250 mM imidazole. Samples from each step were collected and analyzed by Young’s PAGE (4–20%, MOPS, Genscript).

Nsp1 binding kinetics against TAK1 (SinoBiological, #M15-13G) or TRIM21 (MCE, #HY-P71791) were measured by surface plasmon resonance (SPR) on a BIAcore 8K+ (Cytiva). TAK1 was diluted in 10 mM acetate buffer, pH 4.0, and immobilized onto the sensor chip CM5 (Cytiva) at a concentration of 10 ng/μL. Nsp1 was diluted in PBS to different concentrations. Finally, protein concentrations of 81 nM, 162 nM, 324 nM, 649 nM, 1297 nM and 2594 nM were used in the interaction analysis between Nsp1 and TAK1. The detection was performed using a multicycle kinetics method. The contact time ranged from 120 to 150 s, the dissociation time was 240 s, and the flow rate was 30 µL/min.

## Bioinformatics

### Transcriptomic analysis of SARS-CoV-2-infected Calu-3 cells

#### RNA library preparation and sequencing.

Total RNA was isolated from SARS-CoV-2-infected Calu-3 cells using TriPure (Roche, #11667157001) isolation reagent according to the manufacturer’s instructions. mRNA isolation was performed using NEBNext Poly(A) mRNA magnetic beads (NEB, #E7490S) according to the manufacturer’s instructions. RNA libraries for RNA-seq were prepared using an NEBNext Ultra RNA Library Prep Kit (NEB, #E7530) and sequenced on an Illumina HiSeq platform (Illumina, San Diego, USA), yielding 125 bp/150 bp polyA RNA paired-end chain-specific reads.

#### Data processing.

The raw FASTQ reads were analyzed with FastQC (v0.12.1) [103]. The adapters and low-quality bases were removed, and the first 10 bases were trimmed from both reads (--trim_front1 10 --trim_front2 10) using fastp (v0.21.0) [104]. The trimmed and filtered reads were then rechecked with FastQC (v0.12.1).

The processed clean reads were aligned to the human reference genome GRCh38, Ensembl release 93 using STAR (v2.7.1a) [105] with the following parameters: unique mapping was enforced with --outFilterMultimapNmax 1; alignments were output as coordinate-sorted BAM files with compression (--outSAMtype BAM SortedByCoordinate, --outBAMcompression 9); splice junction calling/filtering thresholds were set using --outSJfilterOverhangMin 12 12 12 12, --alignSJoverhangMin 3, and --alignSJDBoverhangMin 3; and chimeric alignment detection was enabled with --chimSegmentMin 12, --chimScoreMin 2, --chimScoreSeparation 10, and --chimJunctionOverhangMin 12, with chimeric outputs recorded as junctions and in a separate SAM file (--chimOutType Junctions SeparateSAMold). The unmapped reads were retained in FASTQ format for the downstream quality assessment. All the other STAR parameters not explicitly listed above were kept with their default settings.

Gene-level read quantification was performed using the ‘summarizeOverlaps’ function from the GenomicAlignments package (v1.24.0) [106]. Genes with fewer than 10 total raw counts across all samples were filtered out to reduce background noise. Count matrices were normalized in the DESeq2 package (v1.42.0) [107] using the median-of-ratios method to account for the differences in sequencing depth and library composition across all samples.

#### Gene clustering.

Fuzzy c-means clustering was performed using the Mfuzz package (v2.48.0) [36] to identify genes with similar temporal expression patterns. The input matrix was created by extracting normalized counts using the ‘counts’ function (normalized = TRUE) from DESeq2 (v1.42.0) and averaging the values across the biological replicates (n = 3) for each time point (6, 24, 48, and 72 hpi). After the data were converted into an ExpressionSet object, Z-score scaling was applied using the ‘standardize’ function to normalize the data, ensuring that the clustering was based on the expression trajectories rather than raw expression values. The ‘mfuzz’ function was subsequently used to assign the genes into clusters. The membership value threshold was set at 0.3, with only genes surpassing the threshold retained for further analysis.

#### Gene ontology enrichment.

Enrichment analysis was performed on gene clusters identified by Mfuzz using the ‘enrichGO’ function from the ClusterProfiler package (v4.9.0.002) [108] and the gene annotations of the org.Hs.e.g.,db database (v3.18.0).

#### Pathway enrichment score.

Immune pathway activity was inferred using pathway-responsive genes for activity inference (PROGENy) [34] of 12 infected samples at 6, 24, 48, and 72 hpi (n = 3). Normalized gene expression values were extracted from the DESeq2 object using the ‘counts’ function (normalized = TRUE) of DESeq2 (v1.42.0). Normalized counts were passed to the ‘progeny’ function of the progeny package (v1.10.0) (scale = TRUE, organism = Human, top = 1000, perm = 1) to obtain the pathway activity scores.

To identify the pathways significantly up- or downregulated at 24/48 hpi, we performed a groupwise comparison using linear modeling. Specifically, samples at 24 and 48 hpi were grouped (n = 6), and samples at 6 and 72 hpi were grouped as a reference group (n = 6). For each pathway, a model was fitted, and the group coefficient t statistic was used to assess the significance of the change and direction of regulation. Positive values indicate higher activity at 24/48 hpi, whereas negative values indicate lower activity at 24/48 hpi.

### Geometric weighted pathway interaction score

To estimate the functional impact of SARS-CoV-2 proteins on host immune-related pathways, we developed the geometric weighted pathway interaction score (GWPIS) framework, an interpretable, protein language model-based method to predict interactions between viral proteins and host pathways.

#### Protein sequence collection.

Full-length amino acid sequences of SARS-CoV-2 viral proteins were retrieved from the NCBI reference genome of the Wuhan-Hu-1 strain (NC_045512.2; https://www.ncbi.nlm.nih.gov/nuccore/NC_045512.2/). The genes involved in immune-related pathways were selected on the basis of the PROGENy[34] dataset, and the corresponding protein sequences of these immune pathway genes were obtained from the Swiss-Prot [109] database (v2025_01).

#### PROGENy-based pathway gene weight extraction and preprocessing.

In this study, pathway gene weights, which refer to the genes whose expression consistently changed when a pathway was activated or inhibited [34], were obtained using the progeny package (v1.10.0) via getModel (organism = Human, top = 1000), matching the parameterization used in the RNA-seq analysis. This setting returns, for each pathway, the top-ranked footprint genes and their corresponding weights. To ensure compatibility with protein-protein interaction analysis in GWPIS, genes without annotated protein sequences in the Swiss-Prot [109] database (v2025_01) were excluded. Given the PPI-based nature of GWPIS, the regulatory direction was not modeled, and PROGENy gene weights were used as absolute values to feature the interaction magnitude.

#### Virus-host pathway protein interaction scoring.

Interaction scores of virus-host protein pairs (SARS-CoV-2 viral proteins vs. human immune-related proteins) were obtained using the sequence embeddings generated by D-SCRIPT (v0.2.8) [33], which employs a pretrained protein-language model to extract contextual features from amino acid sequences and integrates a predicted interresidue contact map to guide a neural network in computing functional interaction scores between protein pairs.

#### Geometric weighted pathway interaction score (GWPIS).

D-SCRIPT [33] outputs a pairwise interaction probability (p ∈ [0–1]) for each virus-host protein pair, representing the likelihood of the association. In GWPIS, these probabilities were used as the interaction scores between the viral protein and each pathway member. For a given pathway, we first computed the interaction scores between the viral protein and all genes assigned to the pathway and then integrated these scores using the pathway gene weights from PROGENy to account for the relative importance of the individual pathway members. This weighting-and-aggregation strategy follows the same general framework as PROGENy, which combines gene-level information with learned weights to summarize pathway-level activity.

We evaluated 3 aggregation methods, arithmetic mean (AM), weighted arithmetic mean (WAM), and weighted geometric mean (WGM), and found that the WGM provided the best performance in the ranking of the positive gene set, with reported roles in regulating NF-κB pathways (S2 Fig and S4 Table). The final geometric weighted pathway interaction score (GWPIS) was computed using WGM as follows:


$$\begin{array}{c} GWPIS\left(P,G_{k}\right)=exp\left(\frac{\sum_{i\in G_{k}}w_{i}\cdot log\left(max\left(p_{i},\epsilon \right)\right)}{\sum_{i\in G_{k}}w_{i}}\right) \end{array}$$

where *G*_*k*_ denotes the set of proteins in immune pathway k, *p*_*i*_ is the predicted interaction score between a viral protein and pathway member *i*, *w*_*i*_ is the PROGENy-derived gene weight, and *ε* is a small constant for numerical stability. This geometric formulation prioritizes high-confidence interactions while accounting for gene importance within the pathway.

### Statistical analysis

All bioinformatics analyses were performed using R (v.4.2.1). For the experiments, graphs were generated using the Prism program (GraphPad software 10; San Diego, CA, USA).

## Supporting information

S1 Table Gene clusters based on expression profiles during host cell infection. (XLSX)

S2 Table Interaction scores of SARS-CoV-2 proteins and host proteins in immune-related pathways. (XLSX)

S3 Table Gene-pathway weight matrix from PROGENy. (XLSX)

S4 Table Positive gene set with reported roles in regulating NF-κB pathway. (XLSX)

S5 Table Interaction scores of SARS-CoV-2 proteins and immune pathways. (XLSX)

S6 Table Sequences of the primers and oligos used in this study. (XLSX)

S7 Table Nsp1-interacting candidates identified by IP-MS. (XLSX)

S1 Fig Expression patterns of genes involved in SARS-CoV-2 infection and screening of nonstructural proteins modulating immune-related pathways (related to Fig 1). **a**: Gene Ontology enrichment analyses of the genes in clusters (C) 1–8 in Fig 1b. The color scale indicates the adjusted p values (P. adjust), and the size of the dots indicates the ratio of genes (GeneRatio) enriched in this pathway. **b**: Gene Ontology enrichment analysis revealed that ‘positive regulation of canonical NF-κB signal transduction’ was significantly enriched only in genes from C1 (red) but not in genes from the other clusters (gray bars). The dashed line represents the threshold of the significant enrichment P value adjusted by Benjamini-Hochberg (B-H) correction (adjusted p = 0.05), and -log10(B-H adjusted p values) are presented.
(TIF)

S2 Fig Benchmarking and validation of GWPIS (related to Fig 2). **a-c**: Distribution of the interaction scores between all SARS-CoV-2 proteins and the NF-κB pathway, calculated using (**a**) arithmetic mean, (**b**) weighted arithmetic mean, and (**c**) weighted geometric mean. SARS-CoV-2 proteins with previously reported immunomodulatory effects on the NF-κB pathway (positive set) are shown in red. **d**: Boxplots showing the rankings of the positive set using the 3 scoring methods. Each dot corresponds to a positive protein highlighted in red in **a-c**; a lower ranking value represents superior model performance in prioritizing these candidates. P values, paired Wilcoxon signed-rank test.
(TIF)

S3 Fig Nsp1 interacts with host TAK1 and TAB1 (related to Fig 3). **a**: MS/MS spectra (red) corresponding to aa 156–172 of TAK1 identified in the IP-MS assay. HEK293T cells were transfected with plasmids expressing FLAG-tagged Nsp1 or a GFP control (Ctrl). Immunoprecipitation was performed using anti-FLAG magnetic beads. **b**: MS/MS spectra (red) corresponding to aa 295–319 (upper left), 337–348 (upper right), 478–489 (bottom left) and 105–115 (bottom right) of TAB1 identified in the IP-MS assay. **c**: HEK293T cells were transfected with a plasmid expressing FLAG-tagged Nsp1 or a GFP control (Ctrl). Immunoprecipitation (IP) was performed using anti-FLAG magnetic beads, and the immunoblots were probed with anti-TAB1 and anti-TAK1 antibodies. FLAG-tagged Nsp1 (red arrowheads) and GFP (green arrowheads) were detected by an anti-FLAG antibody. The results in **c** are representative of two independent experiments.
(TIF)

S4 Fig SARS-CoV-2 Nsp1 inhibits TAK1 downstream pathways (related to Fig 4). **a**-**b**: Western blot analysis showing the phosphorylated and total protein levels of p65, IκBα (**a**), p38 and Erk (**b**) in A549 cells transfected with a plasmid expressing Nsp1 or GFP (Ctrl). The phosphorylated and total protein levels of the proteins were probed with the corresponding antibodies. Left panel, Western blot analysis. Right panel, the relative intensity signals quantified using ImageJ. **c**-**d**: Phosphorylated and total protein levels of p65 and IκBα in HEK293T cells (co-) transfected with plasmids expressing N (**c**), Nsp6 (**d**) or the Nsp1 or GFP control. **e**: Activation levels of the *NECTIN2* promoter. HEK293T cells were first cotransfected with a plasmid expressing a Renilla luciferase reporter (under the control of *NECTIN2*) and a control firefly luciferase in the presence of TAK1-FLAG, TAB1-FLAG or GFP control plasmids; 24 h later, a second transfection of Nsp1 or GFP control plasmids was performed. The cells were collected 24 h later for the dual luciferase assay. Each dot represents a replicate, and the mean and SEM are shown. P values, Mann-Whitney test.
(TIF)

S5 Fig Interaction among the TAK1-TAB1 complex, TRIM21 and Nsp1 (related to Figs 5 and 6). **a**: Relative mRNA levels of TAK1 in N-Caco-2 cells infected with SARS-CoV-2 RDPs at an MOI of 0.001. The mock infection group was cultured with an equal volume of DMEM. β-ACTIN was used as an internal control. Each dot represents a biological replicate, and the mean and SEM are shown. P values, One-way ANOVA test. **b**-**c**: HEK293T cells were first transfected with plasmids expressing HA-Ub and TAK1-FLAG (**b**) or TAB1-FLAG (**c**) for 24 hr and then transfected again with cMYC-tagged Nsp1 or a GFP control (Ctrl) plasmid. Ubiquitinated TAK1/TAB1 was immunoprecipitated (IP) by anti-FLAG magnetic beads, and immunoblots (IB) were analyzed using anti-HA (Ub), anti-FLAG (TAK1/TAB1) and anti-cMYC (Nsp1) antibodies. The relative signals quantified by ImageJ are shown in the right panel. **d**: MS/MS spectra (red) corresponding to 218–234 aa of TRIM21 identified in the IP-MS assay. HEK293T cells were transfected with a plasmid expressing FLAG-tagged Nsp1 or a GFP control (Ctrl). Immunoprecipitation was performed using anti-FLAG magnetic beads. **e**: Co-IP analysis showing that TAK1 binds to the SPRY domain of TRIM21. HEK293T cells were transfected with plasmids expressing TAK1-FLAG and cMYC-tagged full-length (FL)-, ΔSPRY- and SPRY-TRIM21 (shown in Fig 6b) or the GFP control (Ctrl). Immunoprecipitation (IP) was performed using anti-FLAG magnetic beads. TRIM21 (red arrowheads) and GFP (green arrowheads) were detected by the anti-cMYC antibody. **f**: Co-IP analysis showing that TRIM21 binds to the C-terminus of TAK1. HEK293T cells were transfected with plasmids expressing cMYC-tagged TRIM21 and FLAG-tagged FL, N-terminal (1–303 aa, N) or CC-terminal (304–606 aa, C) sequences of TAK1 or the GFP control. IP was performed using anti-cMYC magnetic beads. TAK1 (blue arrowheads) and GFP (green arrowheads) were detected by the anti-FLAG antibody. **g**: Levels of TRIM21 transcripts (relative to β-ACTIN) in HEK293T cells transfected with a lentiviral vector expressing shRNA targeting TRIM21 (shTRIM21) or luciferase (shLuc). P values, Welch’s t test. **h**: HEK293T cells were transfected with a lentiviral vector expressing shTRIM21 or shLuc. After puromycin selection, the cells were transfected with a plasmid expressing cMYC-tagged Nsp1 or a GFP control (Ctrl) for 36 hr. Nsp1 (yellow arrowheads) and GFP (green arrowheads) were detected by an anti-cMYC antibody. The results in **a, b, c,** and **h** are representative of two independent experiments.
(TIF)
